# Dependence of structure and temperature for lithium-rich layered-spinel microspheres cathode material of lithium ion batteries

**DOI:** 10.1038/srep08403

**Published:** 2015-02-12

**Authors:** Di Wang, Ruizhi Yu, Xianyou Wang, Long Ge, Xiukang Yang

**Affiliations:** 1 (Key Laboratory of Environmentally Friendly Chemistry and Applications of Ministry of Education, Hunan Province Key Laboratory of Electrochemical Energy Storage and Conversion, School of Chemistry, Xiangtan University, Xiangtan 411105, China)

## Abstract

Homogeneous lithium-rich layered-spinel 0.5Li_2_MnO_3_·0.5LiMn_1/3_Ni_1/3_Co_1/3_O_2_ microspheres (~1 μm) are successfully prepared by a solvothermal method and subsequent high-temperature calcinations process. The effects of temperature on the structure and performance of the as-prepared cathode material are systemically studied by X-ray diffraction (XRD), scanning electron microscope (SEM), transmission electron microscope (TEM), galvanostatical charge/discharge and electrochemical impedance spectra. The results show that a spinel Li_4_Mn_5_O_12_ component can be controllably introduced into the lithium-rich layered material at 750°C. Besides, it has been found that the obtained layered-spinel cathode material represents excellent electrochemical characteristics. For example, it can deliver a high initial discharge capacity of 289.6 mAh g^−1^ between 2.0 V and 4.6 V at a rate of 0.1 C at room temperature, and a discharge capacity of 144.9 mAh g^−1^ at 5 C and 122.8 mAh g^−1^ even at 10 C. In addition, the retention of the capacity is still as high as 88% after 200 cycles, while only 79.9% for the single-phase layered material. The excellent electrochemical performance of the as-prepared cathode material can probably be attributed to the hybrid structures combining a fast Li-ion diffusion rate of 3D spinel Li_4_Mn_5_O_12_ phase and a high capacity of the layered Li-Mn-Ni-Co-O component.

Lithium-rich cathode materials xLi_2_MnO_3_·(1−x)LiMO_2 _(M = Mn, Co, Ni, etc.) with layered structure have been the focus of intense research interest as one of the most promising cathode materials for high-energy density lithium ion batteries due to their high reversible capacities of up to 250 mAh g^−1^ when cycled above 4.5 V^1^,^2^,^3^, which represents an important milestone in materials design for advanced lithium-ion batteries^3^,^4^,^5^. However, although the discharge specific capacity of these lithium-rich layered cathode materials is higher than that of traditional cathode materials such as LiCoO_2_, Li_2_MnO_4_ and LiFePO_4_ nearly twice as much, their industry application still needs to solve the following technical bottleneck: (1) the huge irreversible capacity loss after first cycle; (2) the poor rate capability; (3) the insufficient cycling performance under the high voltages; and (4) the voltage attenuation caused by the phase transformation during cycling process. The huge irreversible capacity loss has been attributed to the elimination of oxygen atom and lithium ion vacancies from the layered lattice at the end of the first charge^4^, and the poor rate capability could be related to the low electronic conductivity associated with the Mn^4+^ ions and the thick SEI layer formed by a reaction of the cathode surface with the electrolyte^5^,^6^. In order to overcome above problems, in recent years much effort has been devoted to the preparation of the composites that integrate both high power characteristics and rate capability to meet the ever-increasing demand for new cathode materials of lithium ion batteries.

Currently, spinel lithium-ion battery cathodes with three-dimensional (3D) interstitial space for diffusion of lithium ions have also attracted more and more attention due to its high discharge potential and excellent rate capability^7^,^8^. Unfortunately, the spinel cathodes can only deliver a capacity of less than 150 mAh g^−1^, which is far lower than that of the lithium-rich layered cathode materials. Therefore, it is interesting and possible to integrate the lithium-rich layered and spinel cathode materials into a composite, which might combine the merits of the two components, such as high capacity and excellent rate capability. The strategy to design high-performance electrodes by using hybrid layered-spinel structures is firstly adopted at Argonne National Laboratory^9^. Recently, Shi^10^ reported a nanostructured hybrid layered-spinel Li_1.13_Mn_0.75_Ni_0.25_O_2.32_ cathode material, which revealed greatly improved first-cycle efficiency (up to 90%) compared with that of the layered material (71%). Besides, Wu^11^ also reported lithium-rich layered cathode material encapsulated with nano-sized spinel LiNi_x_Mn_2−x_O_4_ or Li_1+x_MnO_4_ membrane for high-energy and high-power lithium-ion batteries. Obviously, lithium-rich cathode materials with layered-spinel hybrid structures are currently becoming the research hotspots. However, it is worth thinking how to integrate the controlled spinel components into the layered materials and what kind of spinel materials will maximally improve the comprehensive performance of composite materials.

Recently, our group has designed and synthesized lithium-rich layered materials with improved electrochemical performances^12^,^13^,^14^. Based on our previous works, herein, we report a homogeneous lithium-rich layered Li-Mn-Ni-Co-O microsphere combined with a new spinel Li_4_Mn_5_O_12_ component via a solvothermal process and subsequent simple heat treatment. As the the Li_4_Mn_5_O_12_ spinel has been prepared at a low temperature^15^,^16^ and it has a variable structure^17^ which will show a change of structure from Li_4_Mn_5_O_12_ phase to Li_2_MnO_3_ phase when the temperature was raised above 700°C^18^. Therefore this lithium-rich spinel Li_4_Mn_5_O_12_, which has a three-dimensional (3D) interstitial space permiting fast diffusion of lithium ions and stable tetravalent manganese^19^, was controllable introduced into the lithium-rich layered cathode material for the first time. We hope that this hybrid cathode material can maximize the high capacity of the layered component and inherent advantages of the 3D Li^+^ insertion/extraction framework of the spinel component. However, we have found that during the calcinations process the temperature of solid phase reaction will dramatically influence the performance of the material. Thus, in this paper, the effects of preparation temperature on the structure and performance of the as-prepared cathode materials were systemically studied.

## Results and Discussion

**Figure 1:**
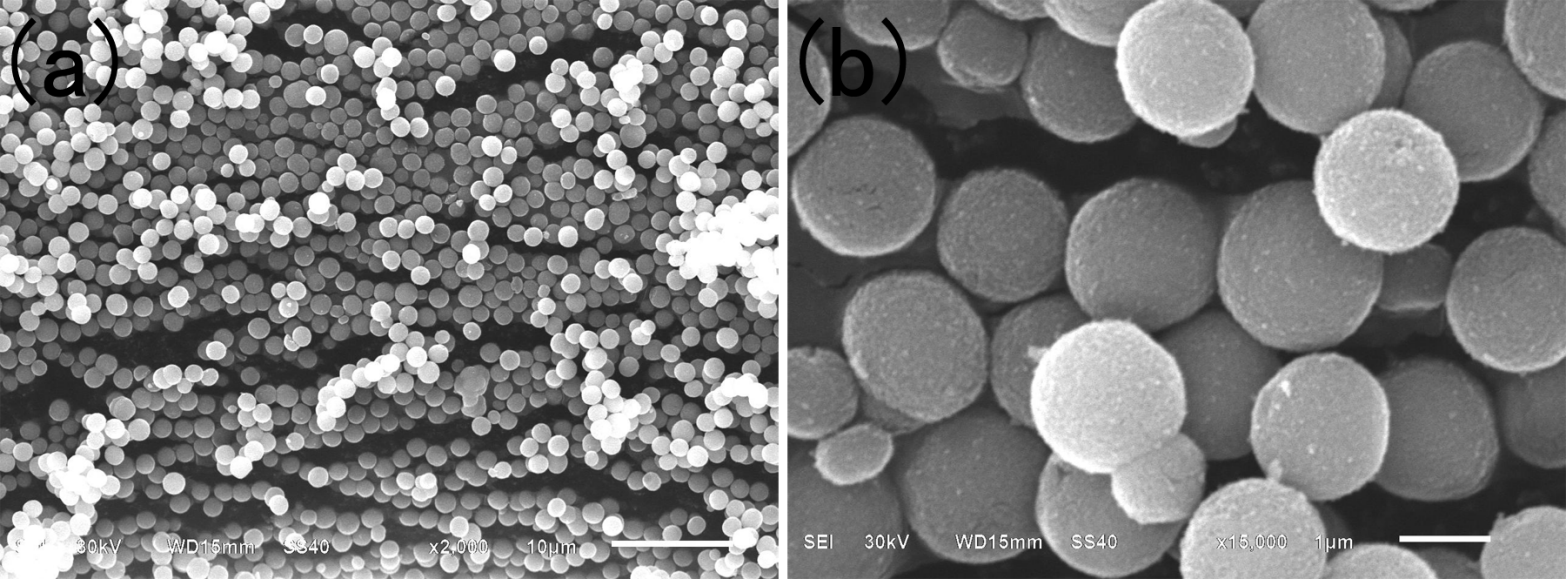
SEM images of the carbonate precursor via the solvothermal method.

**Figure 2:**
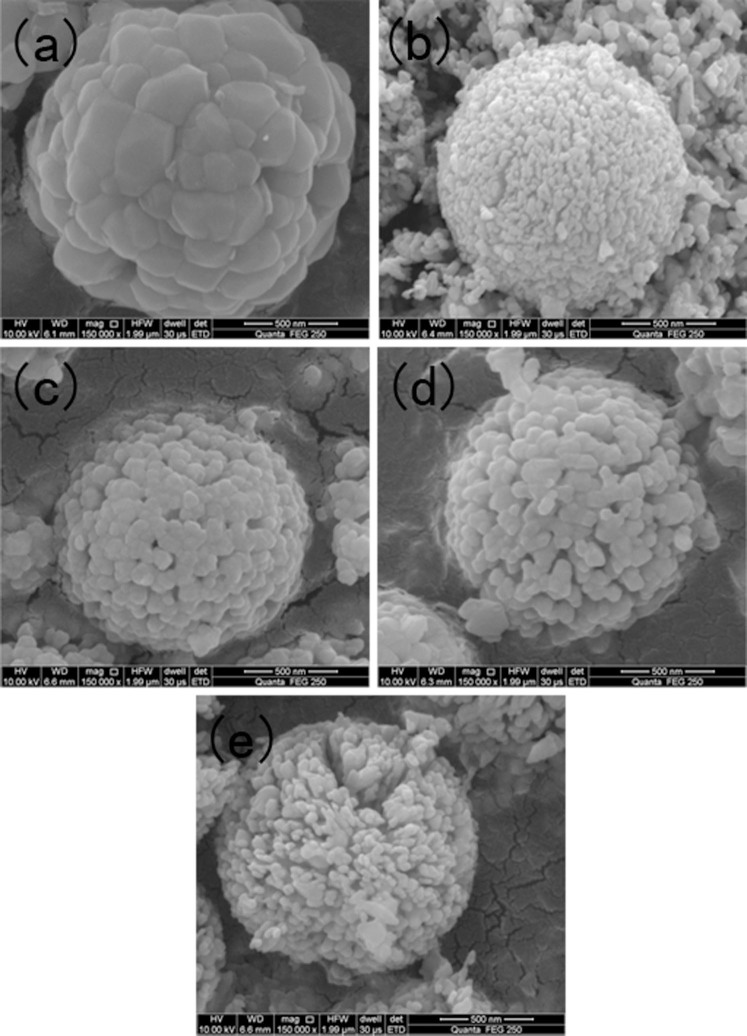
SEM images of the samples calcined at different temperatures: (a) D700, (b) D750, (c) D800, (d) D850, (e) D900.

The typical surfaces and morphologies of the carbonate precursor and corresponding cathode materials prepared under the different calcination temperatures are examined by SEM, as shown in Fig. 1 and Fig. 2, respectively. Fig. 1 shows that the carbonate precursor particles obtained from the solvothermal process are spherical morphology, and the shape of the particle is regular and the size is homogeneous. In addition, the average diameter of the precursor particles is about 1 μm. Besides, as shown in Fig. 2, after the carbonate precursors were calcined at high temperatures with Li_2_CO_3_, the resultant cathode materials still maintained a good spherical morphology like precursor particles. In addition, each of the spherical cathode particles was made up of a large number of primary grains. A close observation shows that the primary grains of the samples are strongly influenced by the calcinations temperatures. It can be seen from Fig. 2(a) that the sample calcined at 700°C (denoted by D700 ) has a clear contour of cubic primary particles with about 250 nm in size which accumulate closely on the surface. When the calcinations temperature was above 700°C, the obtained materials displayed a peeling pomegranate appearance and became obviously smaller size than D700 sample. In addition, the granular shapes of the primary particles slightly protrude toward the outside of the secondary structure of particles along with the temperature increasing.

**Figure 3:**
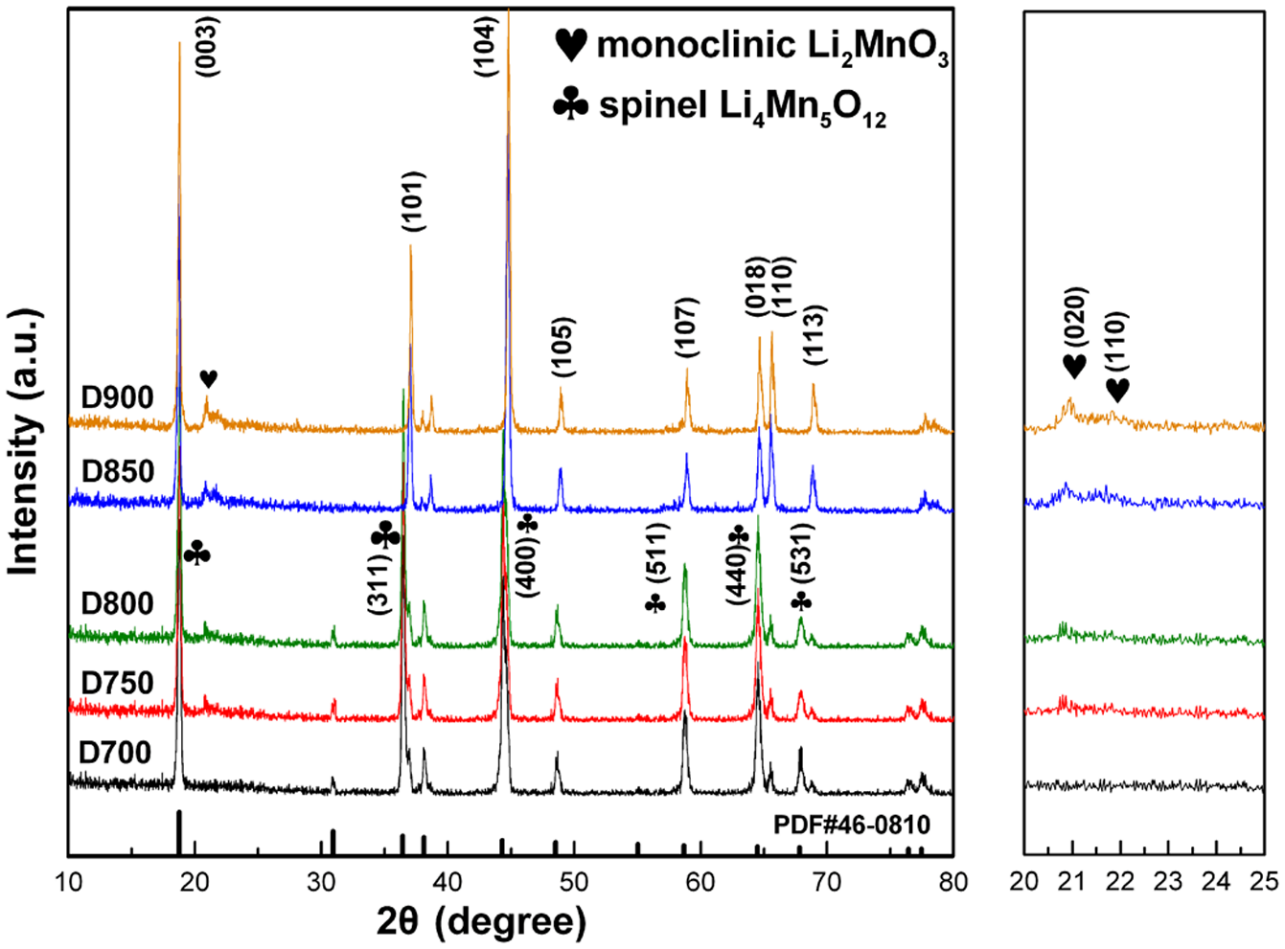
XRD diffraction patterns of the D700, D750, D800, D850 and D900 samples.

The X-ray diffraction (XRD) patterns of as-prepared spherical cathode materials calcined at various temperatures are shown in Fig. 3. All diffraction peaks are quite narrow and sharp, which indicates a high crystallinity of the cathode materials. It can be seen from the Fig. 3 the XRD patterns of the samples calcined at 750°C and 800°C present a layered (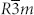 and *C*2*/m*) and spinel hybrid structure. The spinel peaks can be well indexed to cubic Li_4_Mn_5_O_12_ with space group of *Fd3m* (PDF#46-0810) marked as plum blossom pattern in Fig. 3. Additionally, it can be clearly seen that the diffraction peaks of the monoclinic Li_2_MnO_3_ (*C*2*/m*) between 20° and 25° become more clear accompanied with the increasing amounts of the layered phase (from D750 to D800). When the calcinations temperature increases, the D900 and D850 samples show a typical α-NaFeO_2_ layered structure (space group 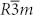), excepting the monoclinic Li_2_MnO_3_ character (space group *C*2*/m*) with some weak peaks between 20° and 25°. Particularly, the clear splitting of the (006)/(012) and (018)/(110) peaks indicates that the two samples have a well-organized layered structure^20^. The XRD patterns of the D900 and D850 samples are also consistent with other lithium-rich layered cathode materials^21^. When the calcination temperature further decreased to 700°C, the obtained D700 sample shows a hybrid structure between spinel Li_4_Mn_5_O_12_ (*Fd3m*) and layered phases (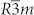). Surprisingly, the weak peaks of the Li_2_MnO_3_ (*C*2*/m*) between 20° and 25° disappear for the D700 sample. The above results demonstrate that the annealing of Li_4_Mn_5_O_12_ at the temperature above 700°C leads to existence of monoclinic Li_2_MnO_3_, which is in consistence with the previous reports^16^,^17^,^18^,^19^. In other words, it suggests that the formation of spinel phase can be simply controlled by calcinations temperature, which provides an effective approach for design and development of high-performance cathode materials. As both of D750 and D800 have the two phases (layered and spinel), the ratio of the two phases has been calculated by XRD analysis approach to distinguish the two phases, and the result is showed in Table 1. It can be found that when the temperature is controlled at 700°C, there is no layered phases. When the temperature rises from 700 to 750°C, 16.9% of spinel phase will be replaced by the layered phase, and while temperature rises from 750 to 800°C, 58.91% of the spinel phase will be replaced by the layered phase. Further, when the temperature increases to 850 or 900°C, the spinel phase will disappear and the samples show a typical α-NaFeO_2_ layered structure with monoclinic Li_2_MnO_3_ structure (*C*2*/m*).

**Figure 4:**
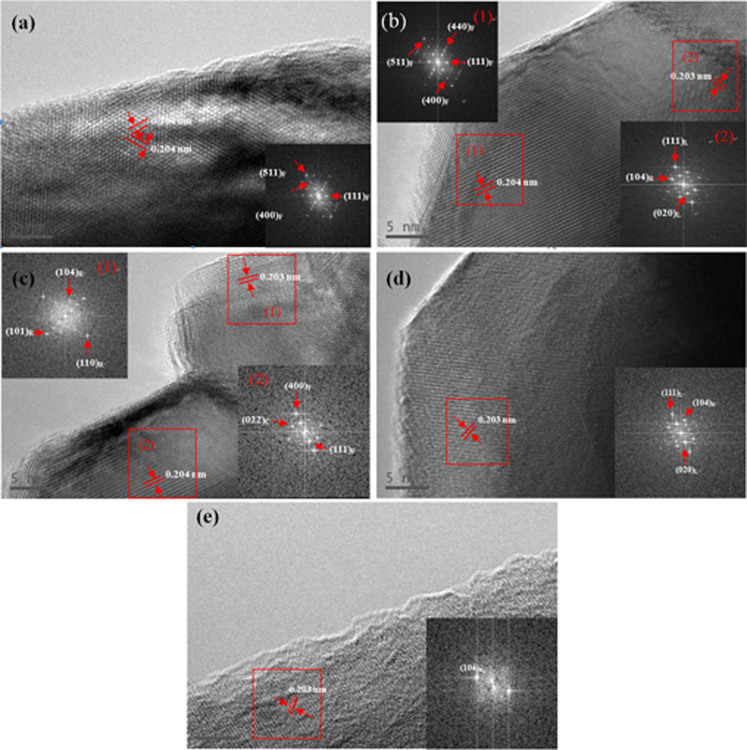
HRTEM images and corresponding FFT of the samples calcined at different temperatures: (a) D700, (b) D750, (c) D800, (d) D850, (e) D900.

The microstructures of the samples are further characterized by high-resolution transmission electron microscopy (HRTEM). The HRTEM images and corresponding fast Fourier transformation (FFT) of the D700 sample is shown in Fig. 4(a), and it can be seen that the diffraction spots can be indexed to Li_4_Mn_5_O_12_ structure for the (*Fd3m*) space group taken from along (400), (511) and (111) directions. From the Fig. 4(b) and (c), both the images of D750 and D800 give two sets of clear lattice fringes with the *d*-spacing of 2.03 A corresponded to (104) plane of α-NaFeO_2_ layered structure and 2.04 A corresponded to (400) plane of (*Fd3m*) Li_4_Mn_5_O_12_ structure. In addition, the D750 sample have spinel and layered crystalline with the characteristic plane (400) and (111) of (*Fd3m*) Li_4_Mn_5_O_12_, the (020), (022) plane of (*C2/m*) Li_2_MnO_3_ and the (104) plane of α-NaFeO_2_ layered structure (space group 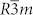), as illustrated in Fig. 4(b). The images of layered D850 and D900 samples are shown in Fig. 4(d) and (e), it can be obviously found that both of the samples consist with the pure characteristic plane (104) of α-NaFeO_2_ (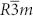). As a consequence, when the calcinations temperature is controlled at 750°C and 800°C, the as-prepared materials show a combination structure of lithium-rich layered and spinel Li_4_Mn_5_O_12_ phases. However, when the samples are calcined below 750°C, the as-prepared materials show only a spinel Li_4_Mn_5_O_12_ structure. Moreover, the as-prepared materials just show a pure lithium-rich layered structure when calcined above 800°C. These consequences are in good agreement with the XRD results in Fig. 3.

**Figure 5:**
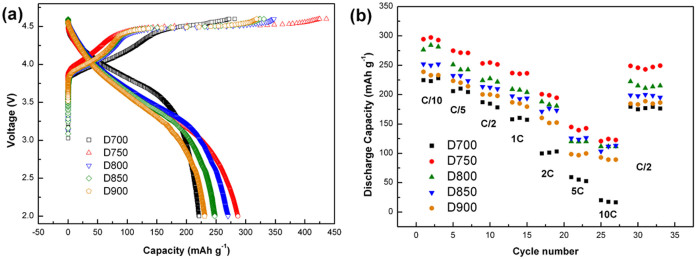
(a). First charge/discharge profiles of D700, D750, D800, D850, D900 cells between 2.0 and 4.6 V at a rate of 0.1 C. (b). Capacity retention of D700, D750, D800, D850 and D900 during cycling between 2.0 V and 4.6 V at various rates from 0.1 C to 10 C.

To compare the electrochemical performance of the as-prepared materials, coin cell tests are performed using the D700, D750, D800, D850 and D900 samples as the cathode materials and Li metal as a counter electrode. Fig. 5 displays initial charge/discharge profiles of the cells between 2.0 V and 4.6 V at a rate of 0.1 C (1 C corresponds to 200 mA g^−1^ current density). The D750, D800, D850 and D900 samples show a representative profile of lithium-rich layered materials with a sloping curve below 4.5 V and a long plateau around 4.5 V in the first charge process. The sloping curve below 4.5 V corresponds to lithium ions extraction from the layered LiMO_2_ component with the concomitant oxidation of Ni^2+^/Ni^4+^ and Co^3+^/Co^4+^, while the long plateau ~4.5 V is consistent with the removal of lithium ions and oxygen from the Li_2_MnO_3_ component^23^,^24^. In addition, the D700 sample also shows a short plateau around 4.5 V, which indicates that a small amount of Li_4_Mn_5_O_12_ had been decomposed into monoclinic Li_2_MnO_3_ when calcined at 700°C^22^. As shown in Fig. 5(a), the D750 cell delivers a highest initial discharge capacity of 289.6 mAh g^−1^, whereas the D700, D800, D850 and D900 cells exhibit an initial discharge capacity of 221.2, 270.104, 247.54 and 230.757 mAh g^−1^, respectively.

The rate capabilities of these cathode materials are shown in Fig. 5(b). Compared D700 with D750, it can be found that the rate performance is well improved when the calcinations temperature increased to 750°C. The D750 cell delivers a discharge capacity of 254.8 mAh g^−1^ at 0.5 C, 144.9 mAh g^−1^ at 5 C and 122.8 mAh g^−1^ even at 10 C. However, the discharge capacity of D700 material at 0.5 C, 5 C and 10 C are only 180.2 mAh g^−1^, 60.7 mAh g^−1^ and 33.2 mAh g^−1^, respectively. Besides, the D800, D850 and D900 have a better rate performance than D700 but doesn't appear as much improvement as D750 materials. Although the D750 and D800 both have the lithium-rich layered and spinel Li_4_Mn_5_O_12_ phases, the rate performance is improved more obviously when the calcinations temperature is controlled at 750°C.

**Figure 6:**
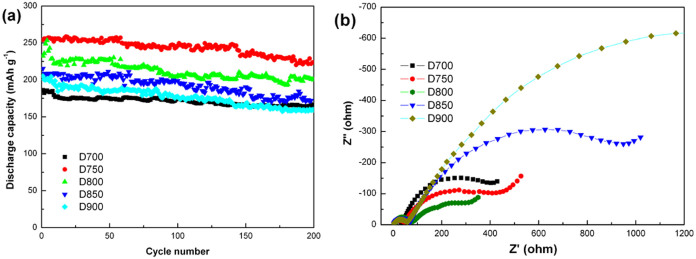
(a). Cycling performance of the samples between 2.0 and 4.6 V at 0.5 C. (b). Electrochemical impedance spectra (EIS) of the D700, D750, D800, D850 and D900 samples.

The cycling performance of the five samples is shown in Fig. 6(a). The spinel samples of D700 exhibits the highest capacity retention of 88.9% with the discharge capacity of 165 mAh g^−1^ at 0.5 C after 200 cycles. Meanwhile, the discharge capacity of D750 reaches 223.6 mAh g^−1^ at 0.5 C after 200 cycles between 2.0 and 4.6 V with a capacity retention of 88%, which has been significantly improved with the introduction of spinel structure^25^. The discharge capacity of D800 decreases to 200.9 mAh g^−1^, and the capacity retentions is 86.5% after 200 cycles. Whereas, the discharge capacities of D850 and D900 are just 172.1 and 159.7 mAh g^−1^ with the capacity retentions 79.9% and 77.9%,respectively. Table 2 summarizes the battery performance of the samples prepared at different temperatures. It can be clearly seen that the mixed phase structure materials (D750 and D800) show relatively high cycling performance compared with those (D850 and D900) with layered structure, which is attributed to the introduction of the spinel structure.

**Figure 7:**
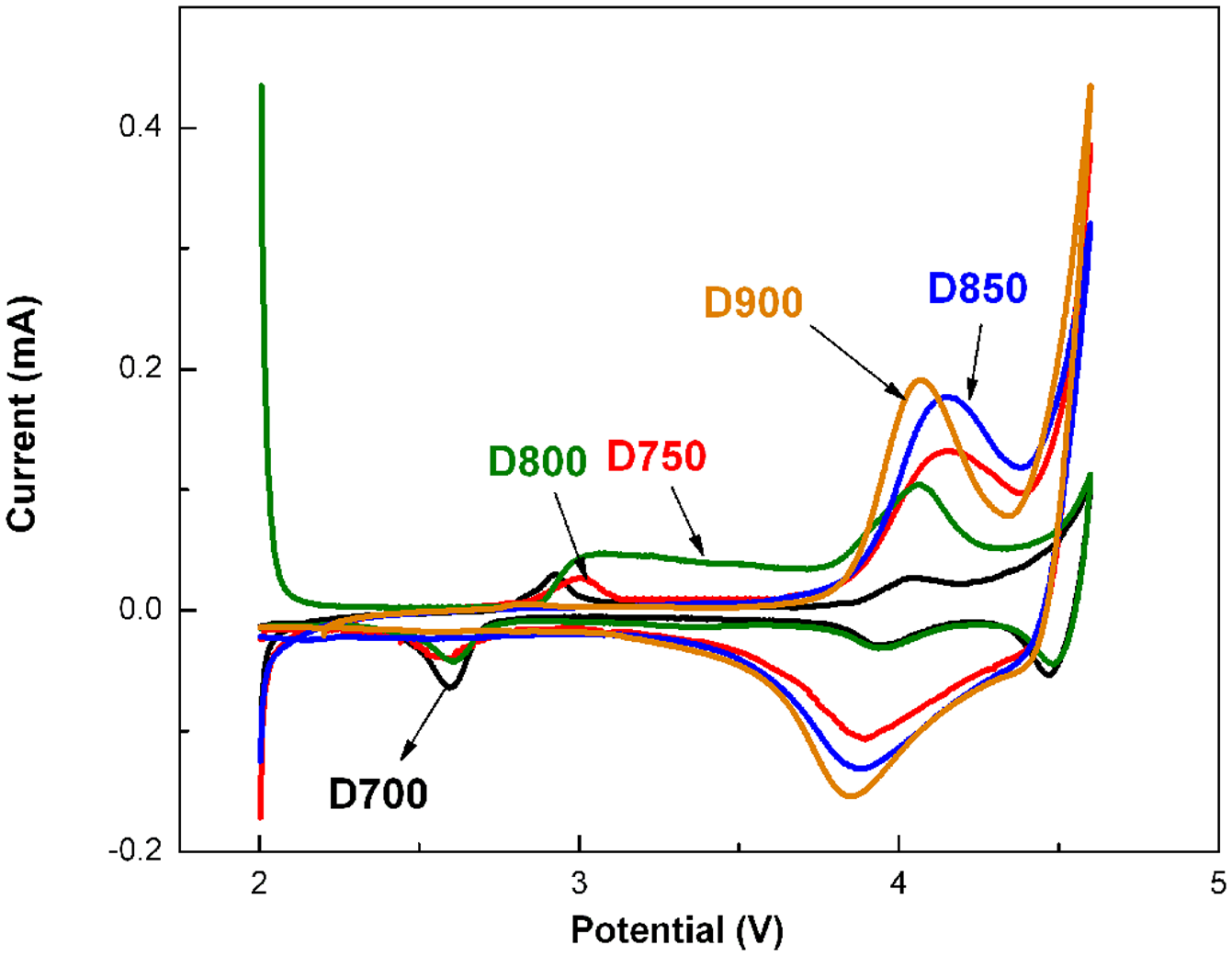
C-V profiles of Li cells with samples of D700, D750, D800, D850 and D900.

To explain the different electrochemical properties of the samples prepared at different temperatures, electrochemical impedance spectroscopy (EIS) is carried out after being charged to 4.6 V. The corresponding Nyquist plots are given in Fig. 6 (b). It can be seen that each plot exhibits two semicircles and one slope line. Usually, the first semicircle in the high frequency region is associated with the resistance (*R*_sf_) due to lithium ion diffusion in the surface layer (including SEI layer and surface modification layer)^26^, the second semicircle in the medium-to-low frequency region is related to the charge transfer reaction between the surface film and the active cathode mass^27^, and the slop line in the low frequency region corresponds to lithium ion diffusion in the material^28^.As the intercept of the first semicircle with the real axis (Z′) refers to the ohmic resistance and the diameter of the second semicircle represents the charge transfer resistance (*R*_ct_)^29^, it can be found that the five samples show approximately ohmic resistance but different charge transfer resistances. The charge transfer resistances of D700, D750 and D800 get somewhat lower compared with D900 and D850. In addition, it can be seen from Fig. 7 that the samples of D800 and D750 shows a anodic peak at about 2.8 V probably identified as Mn^4+^/Mn^3+^ redox couple, while the cathode peaks at about 2.6 V match along with Mn^4+^/Mn^3+^ redox. The reaction at ~2.8 V is associated with the transformation of cubic to tetragonal phase during the extraction of lithium ions from empty 16c octahedral site of the cubic spinel structure^30^. Both of D850 and D900 show an irreversible oxidation peak at ~4.5 V which is correspond to the net loss of Li_2_O and another peak at ~4.0 V which is roughly correspond to the expected value for Ni^2+^/Ni^4+^ reaction. The peak at ~3.3 V indicates that the lithium ion will insert into the MnO_2_ which is derived from Li_2_MnO_3_ at 3.3 V (Mn^4+^ to Mn^3+^)^20^.

GITT is considered to be a reliable method to determine the diffusion coefficient of lithium ions (*D_Li_*) with greater accuracy for compounds with varying composition or voltage, which has been extensively used to calculate the value of *D_Li_* in electrode materials^31^,^32^. The lithium ion diffusion coefficient can be calculated by using the following equation^33^.
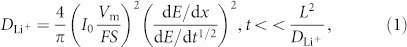
where *V*_m_ is the molar volume of Li_1.5_Ni_0.2_Co_0.2_Mn_0.6_O_2_, which is 20.42 cm^3^mol^−1^ deduced from the crystallographic data. *S* is the active surface area of the electrode, and *L* is the thickness of the electrode^34^. To relax the cell voltage to the steady state, a small current flux and a long time interval should be used for the GITT experiment. In the five samples, both D750 and D800 show the layer-spinel structure, so it is necessary to compare the GITT test results of the two samples.

**Figure 8:**
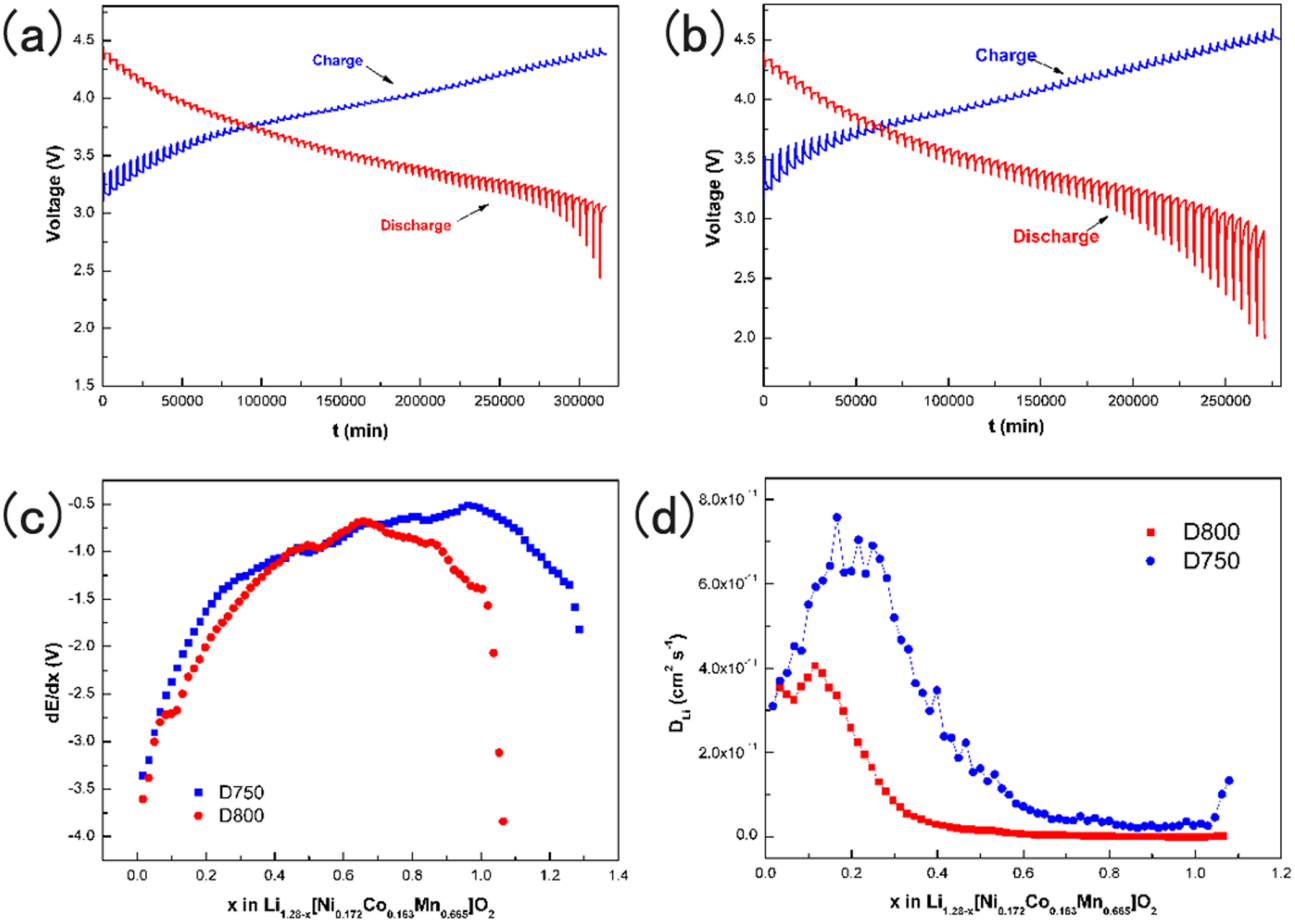
The GITT curves of (a) D750 and (b) D800 as a function of time between 2.0 and 4.6 V; (c) dE/dx and (d) the calculated DLi from the GITT data for the D750 and D800 as a function of the stoichiometry x.

Fig. 8(a) and (b) show the GITT curves of D750 and D800 samples at room temperature, the cell was subjected to charge-discharge at 0.05 C in the voltage window of 2.0–4.6 V. Then the cell was charged at a constant current C/10 rate (1 C was taken as 200 mAh g^−1^) for an interval τ of 10 min followed by an open circuit stand for 1 h to allow the cell voltage to relax to its steady-state value, *E*_s_. The procedure was repeated for the voltage window of operation 2.0–4.6 V. It can be seen that the cell voltage stabilizes to a stable value after the 1 h open-circuit stand after each current flux. At the same time, it can be found that the discharging time of D750 is much longer than the others, which indicates that the voltage drops more slowly during the discharge process and this is accord with the result of charge-discharge test. From Fig. 8(c) it can be seen that variation of d*E*/d*x* about the two samples following the *x* is obvious, in the process of de-insertion the lithium, along with the increasing of x the value of d*E*/d*x* gradually rise, but the d*E*/d*x* of D800 reaches a maximum and then drops with x = 0.63, this phenomenon occurs more early than the samples of D750, this is a strong evidence that the samples can release more capacity during the discharge. Fig. 8(d) shows the *D*_Li_ values of D750 and D800 samples, it is found that the *D*_Li_ values of D750 and D800 are from 4.0 × 10^−11^ to 8.0 × 10^−11^ cm^2^ s^−1^ and from 2.0 × 10^−11^ to 4.0 × 10^−11^ cm^2^ s^−1^, respectively. It is obvious that the the *D*_Li_ values of D750 is slightly higher than that of D800 sample, therefore the improvement of the lithium-ion diffusion coefficient can also be an explanation of the greatly improved electrochemistry performance.

Finally, it should be also pointed out that despite of many studies on this cathode material have been conducting so far, further intensive studies are still necessary. In this paper, the uniform microspheres with layered-spinel composite structure have been synthesized and show an excellent electrochemical performance. Wang' Group^35^ has also reported an layered Li[Li_0.2_Mn_0.54_Ni_0.13_Co_0.13_]O_2_–spinel LiMn_1.5_Ti_0.5_O_4_ composite cathode material, but the particle-size of the samples was not uniform and the initial discharge capacity is just 220 mAh g^−1^, which is apparently much lower than the herein 289.6 mAh g^−1^. In addition, a spinel-layered Li-rich Li-Mn-Co-O material synthesized by Li' Group^36^ showed a uniform microspheres morphology, however the capacity retention at 50 cycles was reported.

In summary, lithium-rich layered-spinel composite cathode materials were synthesized via a solvothermal process followed by calcinations at 750°C. The calcination temperature will critically influence the crystal structure of the obtained cathode material. When the calcination temperature is equal to 700°C, the obtained material shows only a spinel structure; while the calcination temperature is above 850°C, the as-prepared material shows just a lithium-rich layered structure. Besides, the introduction of the spinel structure into the layered lithium-rich cathode materials can effectively improve the electrochemical performance of lithium-rich layered cathode material. Both of the materials obtained at 800 and 750°C show the two phases with different ratios, and we further compared the electrochemical performance of the two samples. The hybrid structure cathode material obtained at 750°C can deliver an initial discharge capacity of 289.6 mAh g^−1^ between 2.0 V and 4.6 V at a rate of 0.1 C, and the discharge capacity at 0.5 C, 5 C and 10 C reached 264.8 mAh g^−1^, 144.9 mAh g^−1^ and 122.8 mAh g^−1^, respectively. Additionally, the capacity retention is still 88% after 200 cycles when cycled at 0.5 C. The *D*_Li_ (GITT) values of the D750 sample are stable at a value of ~6 × 10^−11^ cm^2^/s in the voltage range of 2.0–4.6 V. The charge transfer resistances are obviously reduced with the emergence of spinel structure. Therefore, the lithium-rich layered-spinel composite cathode material prepared at 750°C will possess enormous application prospect in the lithium ion batteries.

## Methods

### Synthesis of Materials

Firstly, 2.0 m mol MnCl_2_·4H_2_O was dissolved in 50 ml ethanediol. Then, 0.498 m mol CoCl_2_·6H_2_O and 0.498 m mol Ni(NO_3_ )_2_·6H_2_O was added to the solution which was stirred vigorously for 0.5 h in the Teflon wares. At the same time, the prepared NH_4_HCO_3_ solution was dropwise added into the mixed solution. Next, the sealed Teflon ware was put into a stainless steel autoclave, which was hold at 200°C for 20 h, followed by cooling at the room temperature to obtain purple precipitates. The precipitates were centrifugalized and washed by ethanol several times, the residue was dried at 60°C. Finally, the as-produced carbonate precursor was preheated at 500°C for 6 h, and mixed with Li_2_CO_3_, then calcined at 700°C, 750°C, 800°C, 850°C, 900°C for 10 h in air respectively, to form the spherical Li-rich cathode materials. To distinguish the products preferably, five kinds of compounds have been named as D700, D750, D800, D850, and D900, which correspond to reaction temperatures of 700°C, 750°C, 800°C, 850°C, and 900°C, respectively.

### Characterizations and Measurements

The structures of samples were characterized by X-ray diffraction on a Rigaku D/MAX-2500 powder diffractometer at 40 kV and 100 mA using a graphite monochromatic and Cu-Ka radiation (k = 0.15418 nm) operated with a scan rate of 2° min^−1^ in the 2*θ* range of 10°–80°. The morphologies were investigated by a JEOL JSM-6610LV scanning electron microscope. Transmission electron microscope JEOL JEM-2100F with accelerating voltage of 200 kV was used in order to obtain TEM images.

The cathodes for testing cells were fabricated by mixing the cathode materials, carbon black, and polyvinylidene fluoride (PVDF) binder with a weight ratio of 80:10:10 in N-methyl pyrrolidinone, which were then pasted on aluminum foil followed by drying under vacuum at 110°C for 24 h. The testing cells were assembled with the cathodes thus fabricated, metallic lithium anode, Celgard 2300 film separator, and 1 M LiPF_6_ in 1:1 ethylene carbonate (EC)/dimethyl carbonate (DMC) electrolyte. The assembly of the cells was carried out in an argon-filled glove box, where water and oxygen concentration were kept less than 5 ppm. All the cells were allowed to age for 12 h before testing. Charge-discharge measurements were performed at room temperature under different rates in a voltage range of 2.0–4.6 V on the Neware battery test system.

The galvanostatic intermittent titration technique (GITT) was performed at room temperature under a small current flux of 0.1 C and a long time interval of 60 min in a voltage range of 2.0–4.6 V on the Neware battery test system.

## Author Contributions

D.W. and X.Y. designed and coordinated the experiments; D.W. and R.Y. carried out the experiment; D.W. and L.G prepared the experimental data; D.W. and X.W. wrote the paper.

## Figures and Tables

**Table 1:** The relative mole ratios of spinel and layered phase in D750 and D800

Sample	Spinel phase (*Fd3m*)	Layered phase (*C*2*/m*)	Ratio of S/L
D750	83.10%	16.90%	4.92
D800	41.09%	58.91%	0.70

**Table 2:** Comparison of battery performance of different samples

Sample		D700	D750	D800	D850	D900
Capacity(mAh g^−1^)	First cycle (0.5C)	186.2	254.1	232.3	215.4	204.9
	200th cycle (0.5C)	165.5	223.6	200.9	172.1	159.7
Capacity fade after 200th cycle (%)		11.1	12.0	13.5	20.1	22.1
